# Cellular and Molecular Profiling of Native Heart Valves in Infective Endocarditis: A Comparative Study with Calcific Aortic Valve Disease

**DOI:** 10.3390/biomedicines14040890

**Published:** 2026-04-14

**Authors:** Anna Sinitskaya, Maria Khutornaya, Alyona Poddubnyak, Maxim Asanov, Alexander Kostyunin, Alexey Tupikin, Marsel Kabilov, Maxim Sinitsky

**Affiliations:** 1 Department of Experimental Medicine, Research Institute for Complex Issues of Cardiovascular Diseases, 650002 Kemerovo, Russia; 2 Institute of Chemical Biology and Fundamental Medicine, Siberian Branch of the Russian Academy of Sciences, 630090 Novosibirsk, Russia

**Keywords:** infective endocarditis, immunohistochemistry, aortic valves, RNA-seq, transcriptomic signature, bioinformatic analysis, differentially expressed genes, gene ontology enrichment

## Abstract

**Background:** Infective endocarditis (IE) affects both native and prosthetic heart valves, the endocardial surface, as well as cardiac implantable electronic devices. Identifying specific IE biomarkers for its early risk stratification remains crucial, particularly in cases with blood culture-negative endocarditis. **Methods:** Eleven native heart valves obtained from IE and calcific aortic valve disease (CAVD) patients were analyzed. Immunohistochemical analysis of a pan-leukocyte marker (CD45), macrophage marker (CD68), T-lymphocyte marker (CD3), B-lymphocyte marker (CD19), neutrophil myeloperoxidase (MPO), and marker of vascular endothelial cells (CD31) was performed. Differentially expressed genes (DEGs) were identified by whole-transcriptome sequencing; proteomic profiling was performed by dot-blotting. **Results:** The immunophenotyping demonstrates the infiltration of macrophages and neutrophils, as well as occasional T-lymphocytes in the IE-affected aortic valves, and the CAVD-affected heart valves were characterized by the absence of neutrophils. For the whole-transcriptome sequencing, 157 DEGs were identified: 124 DEGs were upregulated, and 33 genes were downregulated in the IE-affected heart valves compared to the CAVD-affected ones. According to the dot-blotting, 35 cytokines were identified in the studied heart valves, but only 21 molecules were expressed in both IE and CAVD-affected heart valves. Analysis of proteases and their inhibitors allowed the identification of 13 protease molecules and 18 enzyme inhibitor molecules in all examined heart valves. **Conclusions:** The results of the present study can help to improve our understanding of the IE pathogenesis. In addition, we identified the candidate cellular and molecular-genetic features of IE-affected native heart valves.

## 1. Introduction

Infective endocarditis (IE) is a disease predominantly of bacterial origin that affects both native and prosthetic heart valves, the endocardial surface, and cardiac implantable electronic devices. Despite significant advances in diagnostics, the incidence of IE remains relatively high (approximately 10 cases per 100,000 individuals annually) with a tendency to increase in-hospital mortality [1]. In recent years, the IE incidence caused by *Staphylococcus* spp., particularly *Staphylococcus aureus*, and coagulase-negative staphylococci, has increased. Additionally, a shift toward the predominance of enterococci over streptococci as IE etiological agents has been reported [2]. The host’s systemic immune response triggered by bacterial invasion plays a critical role in the IE pathogenesis and largely determines treatment outcomes [3]. Importantly, the nature of the immune response may vary depending on the pathogen colonizing the heart valves. Patients presenting with similar clinical manifestations may exhibit different underlying pathogenic mechanisms [4].

Echocardiography and microbiological examination of blood and valve tissues are the routine diagnostic approaches for IE. However, no specific IE biomarkers have been described [5]. Several potential biomarkers, including erythrocyte sedimentation rate, procalcitonin, C-reactive protein (CRP), rheumatoid factor, and NT-proBNP, have been proposed, but these markers have a limited specificity for IE diagnostics [6]. Independent research groups have identified several key genes involved in IE pathogenesis, including *CD79A*, *CD79B*, *SPI1*, *S100A9*, *S100A12*, *S100A11*, and *AQP9*, which participate in B-cell signaling pathways, inflammatory and immune responses, and may serve as potential IE biomarkers [7,8,9]. Despite these findings, the identification of other molecules involved in the IE pathogenesis is of great importance for modern biomedical science.

The absence of a single specific diagnostic marker reflects the heterogeneity of IE pathophysiology [10]. Therefore, the identification of specific IE biomarkers for its early risk stratification remains crucial, particularly in cases with blood culture-negative endocarditis. Moreover, an in-depth study of the molecular mechanisms underlying IE using high-throughput methods can help to improve our understanding of the pathophysiology of this disease and identify new molecular targets for IE therapy. Thus, this study aimed to characterize the cellular and molecular-genetic features of native heart valves affected by IE.

## 2. Materials and Methods

### 2.1. Group Description

Native heart valves affected by IE (case group, n = 6) and calcified aortic valve disease (CAVD) (comparison group, n = 5), obtained during cardiac surgery, were analyzed in the present retrospective study. IE and CAVD were confirmed according to the clinical, microbiological, and echocardiographic data. Blood sampling for IE pathogen identification was performed on all patients included in the case group three times at 6 h intervals, one week before surgery. All IE patients recruited to the study were administered preoperative antibiotic therapy. The inclusion criteria were Caucasian ethnicity, clinically confirmed IE and CAVD, and provided written informed consent to participate in the study. Patients with fever, cancer, and autoimmune diseases were excluded from the study. The additional exclusion criteria for the comparison group were a history of IE, chronic infectious diseases, and drug addiction.

The median age of the recruited patients was 57 and 68 years, and their clinical characteristics and laboratory analysis results are presented in Table 1 and Table 2. This study was approved by the Local Ethics Committee of the Research Institute for Complex Issues of Cardiovascular Diseases (Kemerovo, Russia) (protocol No. 1 dated 26 January 2024) and was conducted in accordance with the standards of Good Clinical Practice and the principles of the Declaration of Helsinki. All patients provided written informed consent to participate in the study. Information about patients included in the presented study was processed in an anonymized form.

### 2.2. Immunohistochemistry

Immunohistochemical analysis was performed on the cryosections of valve tissues placed in Neg-50™ Frozen Section Medium (Epredia, Kalamazoo, MI, USA). Serial cryosections of valve regions with the signs of pathological damage with a thickness of 7 μm were prepared using an HM 525 cryostat microtome (Thermo Fisher Scientific, Waltham, MA, USA). Sections were mounted on microscope slides and subjected to immunohistochemical staining using the Novolink™ Polymer Detection System (Leica Biosystems, Nussloch, Germany) with hematoxylin counterstaining and primary antibodies to the following cellular markers: CD45 (pan-leukocyte marker) (ab10558, Abcam, Cambridge, UK, 1:4000 dilution), CD68 (macrophage marker) (ab227458, Abcam, Cambridge, UK, 1:1000 dilution), CD3 (T-lymphocyte marker, ab16669, Abcam, Cambridge, UK, 1:1000 dilution), CD19 (B-lymphocyte marker) (MA5-32544, Invitrogen, Carlsbad, CA, USA, 1:1000 dilution), and myeloperoxidase (MPO, neutrophil marker) (ab208670, Abcam, Cambridge, UK, 1:4000 dilution). To assess neovascularization, additional staining using antibodies against CD31 (ab9498, Abcam, Cambridge, UK, 1:500 dilution), a marker of vascular endothelial cells, was performed. To assess antibody specificity, positive and negative controls were used. Samples subjected to the standard staining procedure without the addition of primary antibodies served as negative controls. Positive controls for each antibody were selected according to the manufacturer’s specifications.

Prior to staining, tissue sections were fixed in 4% paraformaldehyde for 10 min at room temperature and washed three times with phosphate-buffered saline. Primary antibodies were diluted in 1% bovine serum albumin (Sigma-Aldrich, St. Louis, MO, USA); optimal antibody dilutions were determined empirically. Tissue sections were incubated with primary antibodies overnight at +4 °C. After staining, sections were mounted using Vitrogel mounting medium (BioVitrum, Saint-Petersburg, Russia) and qualitatively analyzed using an AxioImager A1 light microscope (Carl Zeiss MicroImaging GmbH, Jena, Germany) at 400× magnification with transmitted light. Image acquisition and processing were performed using AxioVision software v.4.8 (Carl Zeiss MicroImaging GmbH, Jena, Germany).

### 2.3. RNA Isolation and Whole-Transcriptome Sequencing

For RNA isolation and whole-transcriptome sequencing, fragments of the IE and CAVD-affected heart valves (n = 6 and n = 5, respectively) were placed in TRIzol™ lysis reagent (Invitrogen, Carlsbad, CA, USA) and homogenized using a FastPrep-5G homogenizer (MP Biomedicals, Solon, OH, USA) with Lysing Matrix D tubes (MP Biomedicals, Solon, OH, USA). Total RNA was isolated using the PureLink™ RNA Micro Kit (Life Technologies, Waltham, CA, USA) with simultaneous DNase treatment (On-Column DNase I Digestion Set, Sigma-Aldrich, St. Louis, MO, USA). RNA integrity (RIN) index was assessed using the RNA 6000 Pico Kit (Agilent Technologies, Santa Clara, CA, USA) on the Agilent Bioanalyzer 2100 system (Agilent Technologies, Santa Clara, CA, USA). RNA quantification was performed using NanoDrop^TM^ 2000 Spectrophotometer (Thermo Scientific, Waltham, CA, USA) and Qubit4 Fluorometer (Invitrogen, Carlsbad, CA, USA). RNA integrity numbers (RIN) were above 6 for all samples. Given the nature of clinical valve tissue affected by inflammation and degeneration, this RIN range is considered acceptable for transcriptomic analysis.

Polyadenylated mRNA was purified using NEBNext^®^ Poly(A) mRNA Magnetic Isolation Module (NEB, Ipswich, MA, USA), followed by DNA library preparation using the NEBNext Ultra II Directional RNA Library Prep Kit for Illumina (NEB, Ipswich, MA, USA), combined with NEBNext Multiplex Oligos for Illumina (Unique Dual Index UMI Adaptors DNA Set 1). The DNA libraries’ quality was evaluated using Agilent High Sensitivity DNA Kit (Agilent Technologies, Santa Clara, CA, USA) on the 2100 Bioanalyzer system (Agilent Technologies, Santa Clara, CA, USA). DNA library concentrations were determined by quantitative PCR using the CFX96 Touch Real-Time PCR Detection System (Bio-Rad Laboratories Inc., Hercules, CA, USA). Sequencing was performed on the MGIseq-2000 platform (MGI Tech Co., Ltd., Shenzhen, China) using the FCL PE100 Sequencing Kit with the App-D High-Throughput Sequencing Primer Kit (MGI Tech Co., Ltd., Shenzhen, China) at the SB RAS Genomics Core Facility (ICBFM SB RAS, Novosibirsk, Russia). For the sequencing, the paired-end reads of 100 + 100 bp, ranging from 17 to 56 million paired-end reads, were obtained. Fastq files were deposited to the GenBank under the study accessions PRJNA1449594.

### 2.4. Bioinformatical Analysis of RNA-Seq Data

Bioinformatic analysis was performed using the CLC Genomics Workbench (CLC GW) software v.21.0.5 (Qiagen, Hilden, Germany). The obtained reads were filtered by quality (QV > 20) and length (>15), and adapter sequences were removed. The filtered reads were mapped to the human reference genome (hg38) using Ensembl annotation (v.38.105) with the following parameters: similarity fraction = 0.8, length fraction = 0.8, mismatch cost = 2, insertion cost = 3, and deletion cost = 3. Over 97% of the reads map to the human genome, with 90% of these being exons. The resulting alignments were saved in BAM format.

Differentially expressed genes (DEGs) were identified using multifactorial statistical analysis based on the negative binomial regression by the CLC GW. Statistically significant DEGs were selected based on the following cut-off criteria: |log_2_ fold change| > 1 and FDR (false discovery rate) *p*-value < 0.05. Gene Ontology (GO) enrichment analysis against the GO terms of the biological processes category was performed using the CLC GW with the application of FDR correction. The FDR *p*-value cut-off criteria were assigned as 0.05.

### 2.5. Protein Isolation and Dot-Blotting

Protein isolation from the IE and CAVD-affected heart valves (n = 6 and n = 5, respectively) was performed using T-PER™ Tissue Protein Extraction Reagent (Thermo Scientific, Waltham, CA, USA) supplemented with protease and phosphatase inhibitor cocktails (Thermo Scientific, Waltham, CA, USA). The samples were homogenized in the prepared lysing solution using a FastPrep-5G homogenizer (MP Biomedicals, Solon, USA), followed by centrifugation for 15 min at 10,000× *g*. To remove cellular debris, the resulting supernatant was centrifuged for 30 min at 200,000× *g* and 4 °C on the Optima MAX-XP ultracentrifuge (Beckman Coulter, Brea, CA, USA). Protein concentration was determined by the Pierce™ BCA Protein Assay Kit (Thermo Scientific, Waltham, CA, USA). Semi-quantitative analysis of the key molecules underlying IE pathogenesis (cytokines, chemokines, and proteases) was performed using the Proteome Profiler Human Cytokine Array Kit (R&D Systems, Minneapolis, MN, USA) and the Proteome Profiler Human Protease/Protease Inhibitor Array Kit (R&D Systems, Minneapolis, MN, USA). Signal detection was performed on the Odyssey XF Imaging System (LI-COR Biosciences, Lincoln, NE, USA); based on the positive signal detection, the molecules expressed in the examined heart valves were selected. Densitometry and normalization of dot-blotting results were carried out using ImageJ v.1.15 software (National Institutes of Health, Bethesda, MD, USA) according to the kit manufacturer’s protocol. All samples were analyzed in two technical replicates.

### 2.6. Statistical Analysis

Statistical analysis was performed using GraphPad Prism 8 (GraphPad Software Inc., USA). Laboratory parameters were presented as median (Me) and 25th and 75th percentiles. Differences in the expression level of proteins identified during semi-quantitative analysis were assessed using the nonparametric Kruskal–Wallis test with FDR correction for multiple comparisons.

## 3. Results

### 3.1. Histopathological Examination of Explanted Heart Valves

Histological analysis of the IE-affected native heart valves demonstrates the signs of perifocal fibrinoid necrosis with large-focal stromal sclerosis, diffuse focal moderate round cell infiltration, as well as irregular fibrohyalinosis and mild chronic inflammatory infiltration represented by lymphocytes, plasma cells, and isolated giant macrophages. Moreover, a small amount of organized fibrin masses was observed on the surface of the valves. Meanwhile, the CAVD-affected native heart valves were characterized by collagen fiber degradation and pronounced calcification.

### 3.2. Results of Immunohistochemical Analysis

**Figure 1:**
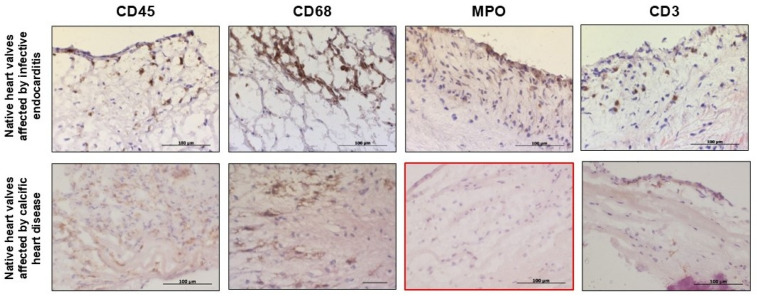
Results of immunohistochemical analysis (400× magnification). No positive staining for neutrophils (MPO) was observed in calcific heart disease-affected heart valves (highlighted in red).

The performed immunophenotyping demonstrates the infiltration of leukocyte-lineage CD45+ cells in both IE and CAVD-affected native heart valves. In the IE-affected aortic valves, inflammatory infiltrates were presented by macrophages (CD68+ cells) and neutrophils (MPO+ cells), as well as occasional T-lymphocytes (CD3+ cells). In the CAVD-affected heart valves, we detected no neutrophils; the obtained immune cells were represented by macrophages and occasional T-lymphocytes (Figure 1).

**Figure 2:**
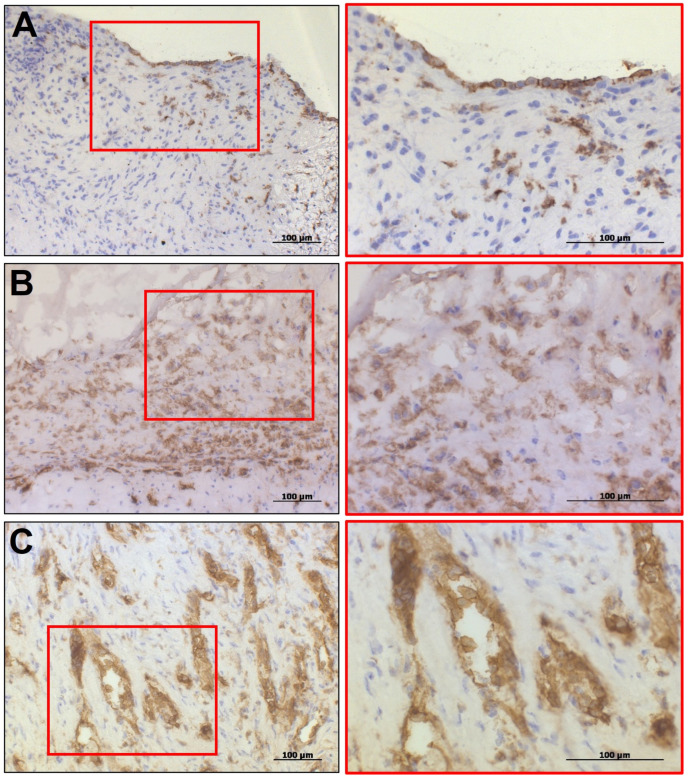
Results of immunohistochemical staining of aortic heart valves to the vascular endothelial cells (CD31) (400× magnification). (**A**) Signs of endothelial-mesenchymal transition in the heart valves affected by calcified aortic valve disease. (**B**) Clusters of CD31+ cells within the extracellular matrix of infective endocarditis-affected heart valves. (**C**) Example of intense neovascularization in the infective endocarditis-affected heart valves. The magnification of the areas of interest is highlighted in red.

The localization pattern of cellular infiltrates differed somewhat between the studied groups. In the IE-affected aortic valves, the obtained immune cells were predominantly localized at the leaflet’s free edge in co-localization with bacterial colonies. In the CAVD-affected heart valves, the large macrophage infiltrates were located within the fibrous layer of the valve at the base and dome of the leaflets, mainly in proximity to calcifications. It should be noted that the occasional CD31+ cells (a marker of vascular endothelial cells) were detected within the fibrous layer in both studied groups. In addition, IE-affected aortic valves were characterized by dense clusters of CD31+ cells within the extracellular matrix and foci of intense neovascularization (Figure 2).

### 3.3. Results of Whole Transcriptome Sequencing

**Figure 3:**
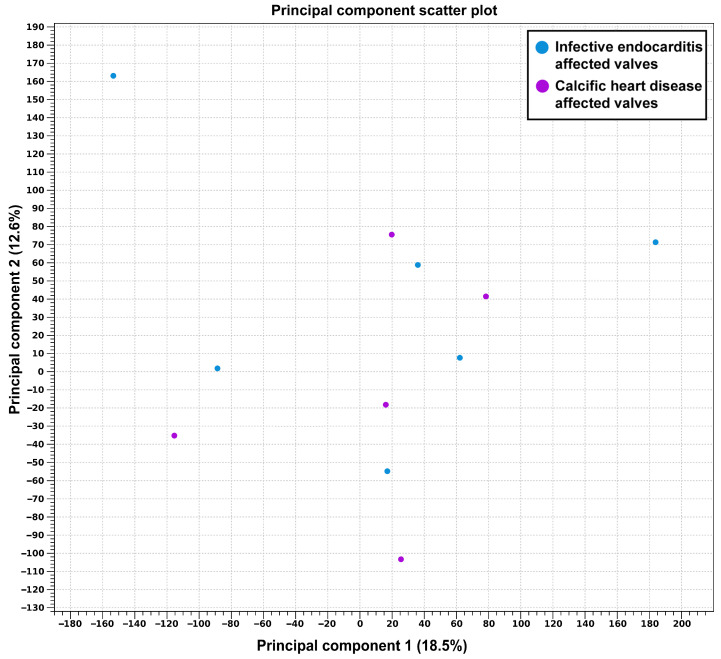
Principal component scatter plot.

Principal component analysis showed that infected native heart valves had a distinct molecular profile compared to the CAVD-affected heart valves (Figure 3).

In total, 157 differentially expressed genes (DEGs) were identified: 124 DEGs were upregulated, and 33 genes were downregulated in the IE-affected heart valves compared to the CAVD-affected ones. For the Gene Ontology enrichment analysis, the identified DEGs were involved in the following biological processes: immune response (enrichment score = 11.46, *p* = 1.51 × 10^−17^), inflammatory response (enrichment score = 8.29, *p* = 4.71 × 10^−13^), humoral immune response (enrichment score: 8.29, *p* = 2.77 × 10^−8^), monocyte chemotaxis (enrichment score = 8.29, *p* = 3.92 × 10^−14^), neutrophil chemotaxis (enrichment score = 8.29, *p* = 9.61 × 10^−19^), lymphocyte chemotaxis (enrichment score = 8.29, *p* = 1.85 × 10^−13^), leukocyte migration (enrichment score = 8.29, *p* = 3.96 × 10^−18^), collagen degradation (enrichment score = 3.21, *p* = 9.92 × 10^−5^), bacterial recognition (enrichment score = 1.54, *p* = 2.90 × 10^−14^), and lipopolysaccharide recognition (enrichment score = 1.54, *p* = 4.65 × 10^−13^).

It should be noted that 15 the most upregulated DEGs (log_2_ fold change > 4.5) were involved in several IE-associated pathogenic pathways, including immune response (*CSF2*, *CSF3*, *CXCL1*, *IL1A*, *IL1B*, *IL24*, *MMP1*, *MMP10*, and *MMP3*), collagen degradation (*MMP1*, *MMP10*, and *MMP3*), extracellular matrix degradation (*MMP1*, *MMP10*, and *MMP3*), cytokine signaling in the immune system (*CSF2*, *CSF3*, *CXCL1*, *IL1A*, *IL1B*, *IL24*, *MMP1*, *MMP10*, and *MMP3*), as well as the innate immune response (*CXCL1*, *IL1B*, *MMP1*, and *CCL17*). The most downregulated DEGs (log_2_ fold change < 2.5) were mainly involved in the following pathways: epigenetic regulation of gene expression (*ADIPOQ*, *CIDEC*, *FABP4*, and *PLIN4*), adipogenesis (*ADIPOQ*, *CIDEA*, and *FABP4*), fibrin clot formation (*CD177* and *VWF*), and lipid droplet organization (*CIDEA* and *CIDEC*) (Table 3).

### 3.4. Results of Proteomic Profiling

According to the dot-blotting, 35 cytokines were identified in the studied heart valves, but only 21 molecules were expressed in both IE and CAVD-affected heart valves. Notably, the IE-affected heart valves were characterized by the increased expression of adiponectin, C-reactive protein, cystatin C, interleukin-8, and lipocalin-2, whereas complement component C5/C5a and complement factor D were downregulated compared to the CAVD-affected heart valves; interleukin-1 alpha and urokinase receptor were unique for the IE-affected heart valves. Analysis of proteases and their inhibitors allowed the identification of 13 protease molecules and 18 enzyme inhibitor molecules in all examined heart valves.

For the semi-quantitative analysis, IE-affected heart valves demonstrated increased expression of ADAM9 (*p* = 0.03) and MMP2 (*p* = 0.01) and the decreased expression of Serpin A5/Protein C Inhibitor (*p* = 0.03), Serpin B6 (*p* = 0.04), and TFPI (*p* = 0.02) compared to CAVD-affected heart valves; CAVD-affected heart valves were characterized by the significantly higher levels of cathepsins (D/S/X/Z/P) (*p* = 0.002, *p* = 0.002 and *p* = 0.03, respectively), cystatins B and C (*p* = 0.01 and *p* = 0.001, respectively) and fetuin B (*p* = 0.001) (Table 4).

## 4. Discussion

IE is an inflammatory disease affecting both the valvular structures of the heart and the endocardium and caused by a variety of pathogens, including bacteria, fungi, and viruses [11]. Despite significant advances in the diagnosis and treatment, IE-associated mortality remains high [12]. Bacterial colonies represented by Gram-positive staphylococci and streptococci, as well as low-virulence bacteria of the HACEK group, attach to the surface of heart valves and form vegetations, which in turn represent one of the causes of embolic complications in IE [13,14]. The immune response to pathogens induces chemoattraction of immune cells, provoking an intense inflammatory reaction and neoangiogenesis [15]. The results of the present study demonstrate histopathological differences between native heart valves affected by IE and CAVD. It was shown that IE-affected aortic heart valves are characterized by aggressive neutrophil infiltration and the presence of foci of neovascularization, in contrast to CAVD-affected valves. Our findings are consistent with the results of other studies [16,17,18].

Today, IE can be relatively effectively diagnosed using the modern instrumental methods and molecular diagnostic techniques; however, their application at the early stages of this disease remains limited, which highlights the need to identify new specific molecules and molecular pathways involved in its pathogenesis [7]. In vitro experiments have shown that monocytes recruited to the damaged valvular structures also participate in the formation of bacterial vegetation by expressing tissue factors and increasing fibrin deposition [19]. Bacterial invasion induces an active inflammatory response in the host, increasing expression of inflammatory cytokines, particularly IL1β and TNFα, which exacerbate endothelial dysfunction through NF-κB-mediated activation of adhesion molecules and matrix metalloproteinases [19,20]. In the present study, upregulation of genes encoding cytokines, including *IL24* (6.91-fold), *IL1β* (4.59-fold), and *IL1α* (4.85-fold), was also observed. According to proteomic profiling, IL1*α* was expressed in IE-affected heart valves only. In the presented study, a relatively large number of upregulated genes involved in the chemotaxis of neutrophils, monocytes, and lymphocytes were identified. These genes encode CXC chemokines (CXCL6, CXCL5, CXCL8, CXCL1, and CXCL3), which are responsible for neutrophil recruitment, and CC chemokines (CCL20, CCL17, CCL3L3, CCL15, CCL7, CCL22, CCL5, CCL3, CCL23, CCL4, and CCL4L2), which are involved in the migration of myeloid cells and T-lymphocytes [21]. It has been shown that CXCL5 and CXCL1 exert pleiotropic effects, including neutrophil activation as well as monocyte adhesion to endothelial cells [20,21]. Interestingly, we found no increased cytokine expression in IE-affected heart valves with concomitant upregulation of inflammatory response genes. This may be explained by preoperative antibiotic therapy in IE patients that can reduce the acute inflammatory infiltrate or alter the expression of proinflammatory molecules at the proteomic but not genomic level. The inflammatory response, which represents an integral component of the IE pathogenesis, disrupts the endothelial barrier of the valve, exposing the underlying vascular structures and thereby leading to activation of the coagulation system and subsequent platelet adhesion, resulting in vegetation formation [22]. It is known that von Willebrand factor (VWF) is expressed in large amounts in the damaged valves and plays a crucial role in bacterial adhesion to both inflamed and damaged endothelium [23]. Notably, in our study, the expression of the *VWF* gene was significantly reduced in IE-affected native heart valves, which, however, does not exclude the possibly increased expression of VWF protein due to the presence of mechanisms of protein post-translational modifications and epigenetic regulations of gene expression.

The development of vegetations on heart valves is also accompanied by remodeling of valve tissues. Experimental animal models have shown that *Staphylococcus aureus* triggers pronounced inflammatory and coagulation responses, as well as excessive extracellular matrix remodeling and collagen degradation, which ultimately leads to destruction of the valvular apparatus [24]. In our study, a significant increase in the expression of matrix metalloproteinase genes *MMP3*, *MMP10*, *MMP1*, *MMP8*, *MMP12*, and *MMP9* was detected in the IE-affected heart valves. However, despite the upregulation of individual proteases, the overall proteomic profiles of both IE and CAVD-affected heart valves exhibited only minor quantitative differences. IE is characterized by focal pathogen-induced lesions of heart valves, initiated either by mechanical damage of the endothelium or by a local inflammatory response, which leads to direct contact between pathogens and the bloodstream as well as with subendothelial components, including extracellular matrix proteins [25]. In particular, in our study, a considerable number of DEGs involved in biological processes such as collagen degradation, extracellular matrix breakdown, and extracellular matrix organization were identified.

It should be noted that the downregulated genes identified in the present study were mainly involved in biological processes such as adipogenesis (*ADIPOQ*, *CIDEA*, and *FABP4*), fibrin clot formation (*CD177* and *VWF*), and lipid droplet organization (*CIDEA* and *CIDEC*). *ADIPOQ*, *CIDEA*, and *FABP4* genes are directly associated with metabolic disorders in individuals with obesity or diabetes mellitus [26,27,28]. *CD177* is predominantly expressed by neutrophils, promoting their migration, activation, and adhesion, as well as the release of inflammatory cytokines under pathological conditions [29]. Literature data indicate high diagnostic significance of this gene for both IE and sepsis [9]. Thus, this molecule may be an important link between metabolic disorders (e.g., obesity) and chronic low-grade inflammation. The downregulation of these genes in the IE-affected heart valves found in the presented study may be associated with a significantly lower number of patients with obesity and metabolic disorders in the case group in comparison with CAVD patients. Generally, lipid accumulation is more characteristic of CAVD than IE [30]. Overall, the identification of potential biomarkers of IE is essential for monitoring disease progression as well as for improving the effectiveness of its treatment. The obtained results can be used to identify new clinical markers for early IE diagnostics and molecular targets for its therapy. This requires validation of the obtained results using routine tests and assessment of the feasibility of using potential markers in peripheral blood samples.

It should be noted that the present study have a number of limitations, namely the use of CAVD-affected native heart valves as the comparison group, the absence of appropriate control group included intact native heart valves and independent validation of RNA-seq results by qPCR, dot-blotting/Western blotting, small sample size, qualitative nature of immunohistochemical analysis and semi-quantitative nature of proteomic analysis, and the possible influence of various confounders (patient heterogeneity, preoperative antibiotic therapy of patients recruited to the study, etc.).

## 5. Conclusions

The results of the immunohistochemical, transcriptomic, and proteomic studies indicate that the IE pathogenesis is based on bacterial invasion, which initiates pronounced neutrophil infiltration of valve tissue, as evidenced by the presence of positive MPO staining exclusively in infected native heart valves. Persistent bacteremia induces continuous stimulation of the immune response, accompanied by increased expression of proinflammatory cytokine genes, as confirmed by transcriptomic sequencing and proteomic profiling data. Furthermore, matrix metalloproteinases significantly contribute to the destruction of heart valves and may also serve as a predictor of systemic complications, including embolic events. The results of the presented study can help to improve our understanding of the IE pathogenesis and develop any innovative strategies for the diagnosis and treatment of this pathology. In addition, we identified a number of important cellular and molecular-genetic features of IE that affected native heart valves. It should be noted that a number of molecules identified in the presented research were not previously described as molecules involved in IE pathogenesis.

## Figures and Tables

**Table 1:** Clinical characteristics of the patients included in the study.

Case	Age	Sex	Dyslipidemia	Obesity	Diabetes Mellitus	Chronic Kidney Disease	Arterial Hypertension	Chronic Heart Failure (NYHA Class)
**Native Heart Valves Affected by Calcified Aortic Valve Disease**								
**1**	66	Male	No	Yes	Yes	No	Yes	II
**2**	68	Female	Yes	Yes	No	No	Yes	III
**3**	72	Female	Yes	Yes	Yes	Yes	Yes	II
**4**	66	Male	Yes	Yes	Yes	Yes	Yes	III
**5**	60	Male	No	No	No	Yes	Yes	II
**Native Heart Valves Affected by Infective Endocarditis**								
**1**	57	Male	No	No	No	No	Yes	II
**2**	34	Male	No	No	No	No	No	II
**3**	49	Male	No	No	No	Yes	No	I
**4**	49	Male	No	No	No	Yes	No	I
**5**	43	Male	No	Yes	No	No	No	I
**6**	61	Male	No	No	No	No	No	II

**Table 2:** Microbiological data and laboratory analysis results of the patients included in the study.

Case/Parameter	Native Heart Valves Affected by Infective Endocarditis						Native Heart Valves Affected by Calcified Aortic Valve Disease				
	1	2	3	4	5	6	1	2	3	4	5
**Microbiological Data (Blood Cultures)**	Sterile	Sterile	*Enterococcus faecium*	*Enterococcus faecium*	Sterile	Sterile	Not applicable				
**Microbiological Data (Heart Valve Biopsy)**	Sterile	Sterile	Sterile	Sterile	Sterile	Sterile	Not applicable				
**Leukocytes, ×10^9^/L**	7.30 [5.40–9.60]						5.25 [4.96–5.77]				
**Neutrophils, ×10^9^/L**	5.35 [2.85–5.50]						2.85 [2.12–3.20]				
**Erythrocyte Sedimentation Rate, mm/h**	7.0 [5.50–28.50]						6.0 [4.25–19.75]				
**C-reactive Protein, mg/L**	2.0 [0.30–11.80]						1.9 [0.70–3.10]				

**Table 3:** Key genes characterized by differential expression in native heart valves affected by infective endocarditis.

Gene Symbol	Gene Annotation	Log_2_ Fold Change	Adjusted *p*-Value
**Upregulated Genes**			
*MMP3*	Matrix Metallopeptidase 3	12.88	3.6591 × 10^−20^
*MMP10*	Matrix Metallopeptidase 10	9.40	3.73219 × 10^−14^
*CXCL6*	C-X-C Motif Chemokine Ligand 6	8.80	3.13956 × 10^−15^
*MT1H*	Metallothionein 1H	8.01	5.46136 × 10^−11^
*CCL1*	C-C Motif Chemokine Ligand 1	7.41	4.43277 × 10^−8^
*CXCL5*	C-X-C Motif Chemokine Ligand 5	7.47	3.98757 × 10^−11^
*IL24*	Interleukin 24	6.91	9.30227 × 10^−9^
*MMP1*	Matrix Metallopeptidase 1	6.81	8.44726 × 10^−10^
*OCSTAMP*	Osteoclast Stimulatory Transmembrane Protein	6.37	1.65535 × 10^−6^
*CSF2*	Colony Stimulating Factor 2	6.11	7.36941 × 10^−7^
*CSF3*	Colony Stimulating Factor 3	5.73	6.09362 × 10^−9^
*CCL17*	C-C Motif Chemokine Ligand 17	5.62	6.09362 × 10^−9^
*CXCL1*	C-X-C Motif Chemokine Ligand 1	5.47	5.9791 × 10^−10^
*IL1A*	Interleukin 1 Alpha	4.85	1.32587 × 10^−8^
*IL1B*	Interleukin 1 Beta	4.59	5.67511 × 10^−9^
**Downregulated Genes**			
*XIST*	X Inactive Specific Transcript	−10.74	3.14614 × 10^−30^
*CD177*	CD177 Molecule	−4.71	2.5156 × 10^−7^
*ADIPOQ*	Adiponectin. C1Q And Collagen Domain Containing	−4.14	2.53325 × 10^−5^
*CIDEC*	Cell Death Inducing DFFA-Like Effector C	−4.03	9.8638 × 10^−6^
*SLC6A3*	Solute Carrier Family 6 Member 3	−3.92	5.00352 × 10^−6^
*PLIN4*	Perilipin 4	−3.51	1.54398 × 10^−6^
*CIDEA*	Cell Death Inducing DFFA-Like Effector A	−3.47	0.0001528
*FABP4*	Fatty Acid Binding Protein 4	−3.41	7.46043 × 10^−7^
*TRIML2*	Von Willebrand Factor	−3.3	0.000308466
*CLEC2L*	CutA Divalent Cation Tolerance Like, Pseudogene	−3.2	1.55422 × 10^−6^
*GPD1*	Tripartite Motif Family Like 2	−3.09	0.000226363
*FHL5*	C-Type Lectin Domain Family 2 Member L	−2.93	9.72922 × 10^−5^
*VWF*	Hemicentin 2	−2.7	0.000336337
*CUTALP*	Glycerol-3-Phosphate Dehydrogenase 1	−2.67	3.12974 × 10^−5^
*HMCN2*	Four And A Half LIM Domains 5	−2.53	3.09976 × 10^−5^

**Table 4:** Densitometric semi-quantitative analysis of proteins expressed in the studied native heart valves.

Analyte	Calcified Aortic Valve Disease-Affected Heart Valves	Infective Endocarditis Affected Heart Valves	Fold Change	*p*-Value
Adiponectin	41,187.024	55,094.823	1.34	0.28
Apolipoprotein A-I	30,714.021	31,592.081	1.03	0.81
Angiogenin	48,184.945	49,910.186	1.04	0.99
Complement Component C5/C5a	37,394.130	25,048.25	0.67	0.22
Chitinase 3-like 1	21,677.389	23,429.946	1.08	0.99
Complement Factor D	22,791.000	12,241.491	0.54	0.13
C-Reactive Protein	31,598.028	51,479.586	1.63	0.21
Cystatin C	12,393.268	19,410.905	1.57	0.99
EMMPRIN	25,334.088	35,131.617	1.39	0.56
ENA-78	3387.074	35,040.66	10.34	0.43
Endoglin	37,747.853	35,302.973	0.93	0.99
IL-8	5032.045	41,844.305	8.31	0.10
Lipocalin-2	22,068.593	34,975.451	1.58	0.22
MIF	30,313.417	31,333.184	1.03	0.80
Osteopontin	36,463.106	35,073.783	0.96	0.99
PF4	39,660.427	27,380.721	0.69	0.99
RANTES	22,652.978	19,405.530	0.86	0.33
RBP-4	41,340.357	39,179.827	0.95	0.80
Serpin E1	36,897.541	33,571.937	0.91	0.70
Vitamin D BP	33,980.459	35,022.642	1.03	0.99
CD31	42,503.153	48,611.201	1.14	0.62
ADAM9	812.52	10,211.06	12.57	0.03
Cathepsin A	47,825.41	35,258.70	0.73	0.21
Cathepsin B	44,374.94	41,363.73	0.93	0.88
Cathepsin D	63,880.25	31,528.17	0.49	0.002
Cathepsin L	50,933.81	35,918.06	0.70	0.35
Cathepsin S	57,217.17	36,953.22	0.65	0.002
Cathepsin V	17,789.07	6779.69	0.38	0.53
Cathepsin X/Z/P	40,234.51	14,120.24	0.35	0.03
Cystatins B	58,585.76	41,873.81	0.71	0.01
Cystatins C	59,150.68	29,077.76	0.49	0.001
Fetuin B	50,737.43	26,778.85	0.52	0.001
DPPIV/CD26	35,531.32	19,175.04	0.53	0.27
MMP-2	6695.38	33,035.01	4.93	0.01
MMP-8	25,881.38	24,074.22	0.93	0.67
MMP-9	37,426.50	50,802.54	1.36	0.69
Proteinase 3	47,987.30	8019.28	0.16	0.66
RECK	29,843.42	13,402.45	0.44	0.13
Serpin A5/Protein C Inhibitor	40,888.21	14,008.94	0.34	0.03
Serpin B6	39,648.96	12,541.21	0.31	0.04
Serpin B8/Proteinase Inhibitor 8	14,193.89	20,183.83	1.42	0.60
Serpin E1/PAI-1	45,972.42	27,998.73	0.60	0.05
Serpin F1/PEDF	50,348.81	38,210.56	0.75	0.13
TFPI	23,944.21	8421.13	0.35	0.02
TFPI-2	22,275.92	5261.98	0.23	0.19
TIMP-1	62,255.59	38,640.94	0.62	0.01
TIMP-2	55,892.73	41,013.36	0.73	0.19

## Data Availability

The raw RNA-Seq data have been deposited in the Sequence Read Archive (SRA) at the National Center for Biotechnology Information (NCBI) under accession number PRJNA1449594.
